# VAAFT: Video Assisted Anal Fistula Treatment; Bringing revolution in Fistula treatment

**DOI:** 10.12669/pjms.315.6836

**Published:** 2015

**Authors:** Mohammad Zarin, Muhammad Imran Khan, Mukhtar Ahmad, Muhammad Ibrahim, Muhammad Asim Khan

**Affiliations:** 1 Dr. Mohammad Zarin, FCPS Gen Surgery (Pak), FMAS (Ind), MRCS (Eng). Consultant Surgeon and Associate Professor, Surgical E Unit Khyber Teaching Hospital Peshawar. Surgical “E” Unit, Khyber Teaching Hospital, Peshawar, Pakistan; 2 Dr. Muhammad Imran Khan, FCPS (Gen Surgery). Consultant Surgeon and Senior Registrar, Surgical E Unit Khyber Teaching Hospital, Surgical “E” Unit, Khyber Teaching Hospital, Peshawar, Pakistan; 3 Dr. Mukhtar Ahmad, FCPS (Gen Surgery). Associate Professor Surgical A Unit, Ayub Teaching Hospital, Abbottabad, Pakistan; 4 Dr. Muhammad Ibrahim, 4^th^ Year General Surgery Resident, Surgical “E” Unit, Khyber Teaching Hospital, Peshawar, Pakistan; 5 Dr. Muhammad Asim Khan, Ist Year General Surgery Resident, Surgical “E” Unit, Khyber Teaching Hospital, Peshawar, Pakistan

**Keywords:** Anal fistula, VAAFT, Meinero Fistuloscope

## Abstract

**Objective:**

To share our findings that the new treatment modality Video Assisted Anal Fistula Treatment (VAAFT) is a better alternate to the conventional treatments of Fistula in Ano in our setup with minor changes in the initial method described by Meinero.

**Methods:**

Karl Storz Video equipment including Meinero Fistuloscope was used. Key steps are visualization of the fistula tract, correct localization of the internal fistula opening under direct vision and endoscopic treatment of the fistula. This is followed by an operative phase of fulguration of the fistula tract using glycine solution mixed with manitol, curetting the tract with curette and fistula brush. Internal opening is closed with a Vicryl 1 suture.

**Result:**

Total of 40 patients were operated using VAAFT from October 2013 to March 2014. Three were re-operated. The other 37 cases were followed up at 6 weeks, 3 months and 6 months. Primary healing took place in 20 (50%) cases at 6 weeks. In the remaining 17 (42.5%) cases, minor discharge occurred with itching which resolved till the next visit at 8 weeks and 12 weeks.

**Conclusion:**

As the main aim in treating fistula is proper identification of the internal opening, excision of the tract and sparing the sphincter function, VAAFT achieves all aims with additional benefits of patients’ satisfaction and negligible scaring.

## INTRODUCTION

Video assisted Anal Fistula treatment (VAAFT) is a minimally invasive technique for complex Anal Fistulas to save the Sphincter damage even after repeated Procedures for recurrences are performed. This technique, initially described by P. Meinero, has been adopted by us as a new promising modality in treating Fistula in Ano. The aim of our study is to share our experience in our setup with minor changes in the initial method described by Meinero P.^1^

The LIFT (Ligations of Intersphinteric Fistula Tract) Procedure^2^ and anal Fistula plug^3^,^4^ procedures are other alternative methods to achieve goals in the management of Fistula in Ano but the drawback of these procedures are need for very high expertise and high cost respectively. On the other hand the conventional Seton, Fistulotomy and Fistulectomy can’t be fully guaranteeing in the treatment of complex fistula in Ano.^1^

## METHODS

Karl Storz Video equipment including Meinero Fistuloscope was used. Key steps are visualization of the fistula tract, correct localization of the internal fistula opening under direct vision and endoscopic treatment of the fistula. This is followed by an operative phase of fulguration of the fistula tract using glycine solution mixed with manitol, curetting the tract with curette and fistula brush. Internal opening is closed with a vicryl 1 suture.^1^ We adopted certain modifications like no use of stapler and synthetic cyanoacrylate because of non-availability and increasing cost of the procedure. Patients were anesthetized with spinal anesthesia and midazolam. Preoperative single dose of 2^nd^ generation cephalosporin was used and 1 dose of the same antibiotic was given post operatively. Post operatively pain killer used were injectable opioids followed by oral pain killers for 3-5 days.

## RESULTS

This procedure was performed for the first time in this institute. We want to share experience of our first 40 cases with mean follow up of 6 months. We operated 40 patients between the month of October 2013 to February 2014 with Video Assisted Anal Fistula Treatment and one case was operated for Video Assisted Pilonidal Sinus Excision. No major complications were noted. Three (7.5%) cases among the 40 cases were re-operated and same technique was advocated. These patients have so far showed promising results. (Table-I)

**Table-I:** Summary of the cases.

Total No. of Patients.	Primary Healing at			Re do Surgery	Complications
	6 weeks	8 weeks	12 weeks		
40	20(50%)	17(40%)	40(100%)	3(7.5%)	None

**Fig.1:**
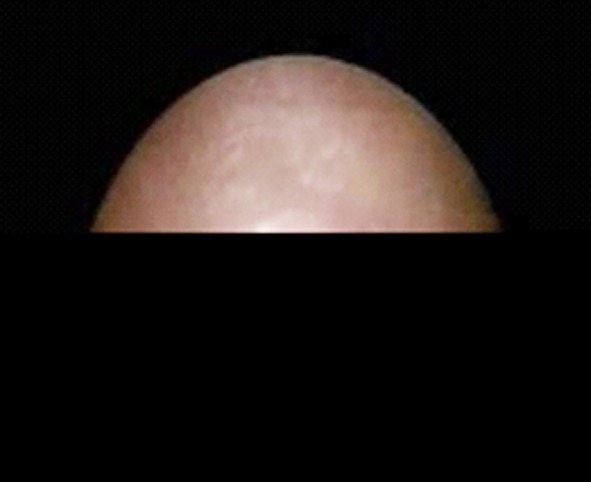
External Fistula Opening.

**Fig.2:**
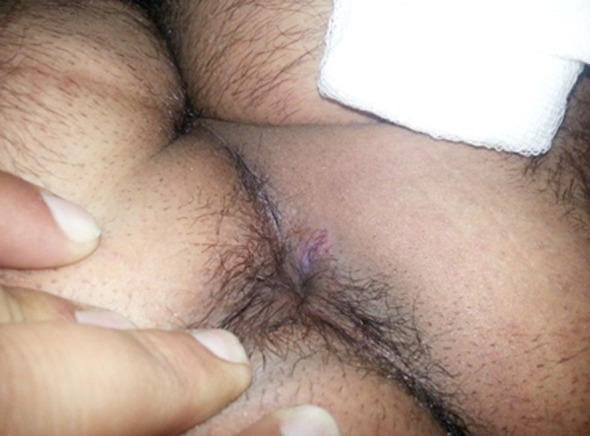
Partial healing after three weeks.

Among the other 37 cases follow up was done at 6, 8 and 12 weeks and primary healing took place (Fig. 1 & 2) in 20(50%) cases at 6 weeks while in the remaining 17 (42.5%) cases minor discharge occurred with itching which resolved till the next visit at 8 weeks and 12 weeks.

Internal Opening was clearly found in 34 cases (85%). In the rest of the cases the tract was identified from the external opening and followed toward the internal opening. The simple straight tract was found in 16 (40%) patients while complex with either multiple tracts of trans- and supra-sphinteric were noted in 24 (60%).

## DISCUSSION

Video assisted Ano rectal Fistula treatment is comparable to all the other procedures done for the Fistula in Ano regarding satisfaction^5^, healing^6^, early recovery because its minimally invasive and allows multiple attempts in case of failure in the first place. The aim in treating fistula in Ano is 3 folds viz identification of the tract, identification of the internal opening and preservation of the anal sphincter.^1^ All these goals are achievable with the VAAFT technique. It can be compared to the gold standard^1^ Fistulectomy and Fistulotomy but the latter are associated with increased trauma and increased morbidity. But the main place where VAAFT fits the most are the Complex and High lying Fistulas where the other competitors like Seton is controversial.^7^,^8^ While in the Endorectal Advancement Flap along with the application of Fibrin glue the success rate is reaching only 54%.^9^ The Ligation of internal Fistula Tract (LIFT Procedure) needs high expertise with success rate ranging from 57% to 95%.^10^-^12^

**Fig.3:**
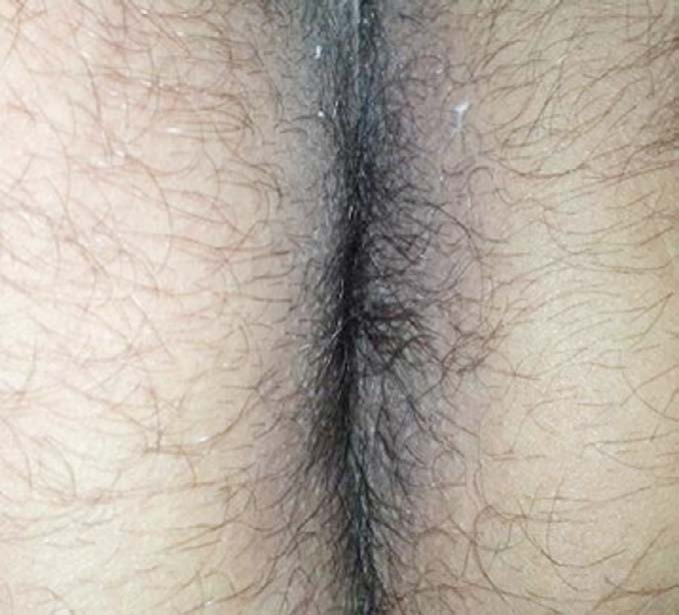
Complete healing after twelve weeks.

Our data appears promising, confirming the results of Ortiz et al. who reported very low recurrence rates after one year using a similar operative principle.^13^

Meinero described successful completion of the method in 100% cases^1^, Schwandner did it in 85% patients^14^, while by Thomas G et al. VAAFT was feasible in 93% of patients.^15^ In our study, we were able to perform the procedure in all of the patients. Internal opening was found in 85% of the patients while G Tomáaš et al. found internal opening in 67% of the patients.^15^

The use of the traditional techniques for the surgical treatment of anal fistulas are associated to a postoperative risk of fecal incontinence up to 45%. VAAFT does not affect fecal continence; however, it may be associated with a recurrence rate of 30%.^16^ There was no procedure-related morbidity in our cohort and also in the cohort of G Tomáaš et al.^15^ although recurrence occurred in 3 cases in 6 months’ observation period in our study. Meinero found 2 cases of post operative urinary retention and scrotal edema in one case possibly due to infiltration of irrigation solution.^1^ No morbidity occurred in Schwandner`s work.^14^ Regarding patients’ recovery, it was more than 87% in case of Meinero^1^, while it was just 67% in case of W piotr.^17^ This difference may correspond to the initial learning curve of the procedure.

Use of vaaft in crohn’s disease by Schwandner O found that there was additional branching in 7/11 operated patients and complete ostium closure in 9 /11 during 9 months’ observation period.^14^ There is no total agreement on a single method of ostium closure. Meinero proposed to close the ostium by stapler but its economically unjustified and its effects are uncertain.^17^

## CONCLUSION

VAAFT is a new modality and our results are comparable to the pioneer of this procedure.^1^ Initial cost of the equipment can be overlooked if the results achieved are over all satisfactory with less chance of recurrence, shortened hospital stay and decreased morbidity.

## References

[1] Meinero P, Mori L (2011). Video-assisted anal fistula treatment (VAAFT): a novel sphincter-saving procedure for treating complex anal fistulas. Techniques in Coloproctology.

[2] Rojanasakul A (2009). LIFT procedure: a simplified technique for fistula-in-ano. Tech Coloproctol.

[3] Lupinacci RM, Vallet C, Parc Y, Chafai N, Tiret E (2010). Treatment of fistula-in-ano with the Surgisis AFP(TM) anal fistula plug. Gastroenterol Clin Biol.

[4] Song KH (2012). New Techniques for Treating an Anal Fistula. J Korean Soc Coloproctol.

[5] García-Aguilar J, Davey CS, Le CT, Lowry AC, Rothenberger DA (2000). Patient satisfaction after surgical treatment for fistula-in-ano. Dis Colon Rectum.

[6] Liu WY, Aboulian A, Kaji AH, Kumar RR (2013). Long-term results of ligation of intersphincteric fistula tract (LIFT) for fistula-in-ano. Dis Colon Rectum.

[7] Ritchie RD, Sackier JM, Hodde JP (2009). Incontinence rates after cutting seton treatment for anal fistula. Colorectal Dis.

[8] Sonoda T, Hull T, Piedmonte MR, Fazio VW (2002). Outcomes of primary repair of anorectal and rectovaginal fistulas using the endorectal advancement flap. Dis Colon Rectum.

[9] Rojanasakul A (2009). LIFT procedure: a simplified technique for fistula-in-ano. Tech Coloproctol.

[10] Rojanasakul A, Pattanaarun J, Sahakitrungruang C, Tantiphlachiva K (2007). Total anal sphincter saving technique for fistulain-ano: the ligation of intersphinteric fistula tract. J Med Asso Thai.

[11] Shanwani A, Nor AM, Amri N (2010). Ligation of the intersphincteric fistula tract (LIFT): a sphincter-saving technique for fistula-in-ano. Dis Colon Rectum.

[12] Bleier JI, Moloo H, Goldberg SM (2010). Ligation of the intersphincteric fistula tract: an effective new technique for complex fistulas. Dis Colon Rectum.

[13] Ortiz H, Marzo M, de Miguel M, Ciga MA, Oteiza F, Armendariz P (2008). Length of follow-up after fistulotomy and fistulectomy associated with endorectal advancement flap repair for fistula in ano. Br J Surg.

[14] Schwandner O (2013). Video-assisted anal fistula treatment (VAAFT) combined with advancement flap repair in Crohn’s disease. Tech Coloproctology.

[15] Tomáaš G, Tomáš S, Oldøich R, Zdenìk K, Beata H, Radoslav H Role of Video Assisted Anal Fistula Treatment in our management of fistula-in-ano.

[16] Mendes C, Ferriera L, Sapocaia R, Lima M, Araujo S (2014). Video-assisted anal fistula treatment: technical considerations and preliminary results of the first Brazilian experience. ABCD Arq Bras Cir Dig.

[17] Walega P, Romaniszyn M, Nowak W (2014). VAAFT: a new minimally invasive method in the diagnostics and treatment of anal fistulas - initial results. Pol Przegl Chir.

